# COVID-19 Pandemic Simulation Modelling in Anaesthesia Residency Training to Predict Delays and Workforce Deficiencies: A Case Study of the Singapore Residency Training Program

**DOI:** 10.7759/cureus.51852

**Published:** 2024-01-08

**Authors:** Lucy J Davies, Christopher Mathew, Ahmad R Pourghaderi, Adeline Xin Yu Leong, Diana Xin Hui Chan, Darren Liang Khai Koh, Addy Yong Hui Tan, Caroline Yu Ming Ong, John Ong, Sean Shao Wei Lam, Sharon Gek Kim Ong

**Affiliations:** 1 Department of Paediatric Anaesthesia, KK Women's and Children's Hospital, Singapore, SGP; 2 Department of Anaesthesiology, Singapore General Hospital, Singapore, SGP; 3 School of Public Health and Preventive Medicine, Monash University, Melbourne, AUS; 4 Department of Anaesthesia, National University Hospital, Singapore, SGP; 5 Department of Anaesthesiology, Intensive Care, and Pain Medicine, Tan Tock Seng Hospital, Singapore, SGP; 6 Department of Gastroenterology, Hepatology, & General Internal Medicine, University of Cambridge, Cambridge, GBR; 7 Singhealth Duke-NUS Academic Medical Centre, Singhealth Duke-NUS Medical School, Singapore, SGP; 8 Department of Anaesthesiology, Sengkang General Hospital, Singapore, SGP

**Keywords:** simulation, modelling, anaesthesiology, residency, pandemic, covid-19

## Abstract

Background

COVID-19 has been the worst pandemic of this century, resulting in economic, social, and educational disruptions. Residency training is no exception, with training restrictions delaying the progression and graduation of residents. We sought to utilize simulation modelling to predict the impact on future cohorts in the event of repeated and prolonged movement restrictions due to COVID-19 and future pandemics of a similar nature.

Method

A Delphi study was conducted to determine key Accreditation Council for Graduate Medical Education-International (ACGME-I) training variables affected by COVID-19. Quantitative resident datasets on these variables were collated and analysed from 2018 to 2021. Using the Vensim® software (Ventana Systems, Inc., Harvard, MA), historical resident data and pandemic progression delays were used to create a novel simulation model to predict future progression delay. Various durations of delay were also programmed into the software to simulate restrictions of varying severity that would impact resident progression.

Results

Using the model with scenarios simulating varying pandemic length, we found that the estimated average delay for residents in each accredited year ranged from an increase of one month for year 2 residents to more than three months for year 4 residents. Movement restrictions lasting a year would require up to six years before the program returned to a pre-pandemic equilibrium.

Conclusion

Systems dynamic modelling can be used to predict delays in residency training programs during a pandemic. The impact on the workforce can thus be projected, allowing residency programs to institute mitigating measures to avoid progression delay.

## Introduction

The COVID-19 pandemic has impacted our lives in many ways. At the height of the pandemic, Singapore adopted a calibrated containment strategy termed ‘circuit breaker’ (CB) [1], marked by strict restrictions on all formal/informal gatherings for a fixed duration. These containment measures also affected local anaesthesiology resident training and progression.

In Singapore, the anaesthesiology training programs are based on the United States of America’s Accreditation Council for Graduate Medical Education-International (ACGME-I) standards. The training comprises five years, of which three years are spent in junior residency (R1, R2, and R3), and two years in senior residency (SR1 and SR2). Besides multispecialty anaesthesia exposure, residents also rotate through sub-specialty postings within different institutions across the country. A sample rotation schedule of the SingHealth Anaesthesiology Residency Program (SHARP) [2], which is the largest anaesthesia training program in Singapore, can be found at "https://tinyurl.com/shanaes".

During the height of the COVID-19 pandemic, movement restrictions between healthcare institutions confined residents to a particular hospital and prevented core subspecialty training (e.g., cardiothoracic, paediatrics, and obstetric anaesthesia) at different locations. This led to progression delays and an inability to present for the post-graduate exams. The fluid nature of these delays, together with recurrent sanctions and changing policies, further exacerbated the difficulty in predicting the progression of affected residents.

Computational modelling is an area of predictive analytics that is increasingly used to improve patient care [3] and transform healthcare education [4]. However, reports on its use to predict challenges in healthcare training are scarce. We, therefore, sought to use simulation modelling to predict the impact of the pandemic on anaesthesia residency training and workforce throughput.

## Materials and methods

Ethics

This study was granted an exemption by the SingHealth Centralised Institutional Review Board (CIRB) (Ref. No.: 2020/2717).

Study design

This is a national, multi-site study involving all three anaesthesiology training programs in Singapore: SingHealth, National Healthcare Group (NHG), and National University Health System (NUHS).

The study consisted of two phases. The first phase involved a Delphi survey [5] to identify the ACGME-I training variables [6] affected by movement restrictions for all programs. The survey involved key institution program stakeholders (program directors, core faculty, and chief residents). Survey questions can be found in Table A1 in the Appendix. We considered a threshold of 80% of agreement for each variable to be included. Using the identified affected training variables from the Delphi survey, de-identified quantitative training data of all residents from 2018 to 2021 were then collated for simulation model input for the second phase of the study. We also compiled data for other training requirements such as the number of case logs and post-graduate examination results.

The second phase of the study involved the development of a system dynamics (SD) simulation model using quantitative resident data obtained from the first phase. SD, a methodology for understanding and managing complex, dynamic systems, is particularly relevant in healthcare and epidemiological contexts. It provides a computer-aided approach for strategy and policy design in these fields, aiding in decision-making processes. The approach uses simulation modelling based on feedback systems theory, which is instrumental in analysing dynamic problems common in healthcare and epidemiological systems. By quantifying interactions within these systems and developing a time-dependent view of their behaviour, SD can reveal less obvious relationships, delays, and unintended consequences of various health policies or epidemiological interventions. This makes it a valuable tool for addressing the complexities inherent in public health and disease management [7]. Our project utilized Vensim PLE, specifically the Vensim 8.1 release, renowned for its efficiency in constructing, simulating, evaluating, and enhancing complex dynamic systems; this version is a key component of the Vensim software suite (Ventana Systems, Inc., Harvard, MA). Of the simulation modelling techniques available, we applied SD modelling as these methods have the ability to capture both detailed system interactions (dynamic complexity) and structure (causal relationships) to provide a risk-free virtualized experimentation platform [8,9].

**Figure 1:**
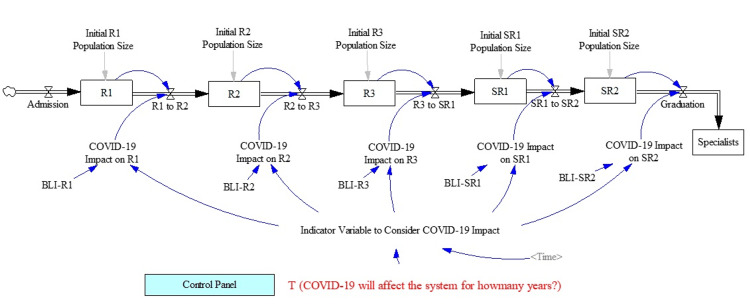
Simulation model framework R1-3: junior residency year 1-3; SR1-2: senior residency year 1-2; BLI: baseline impact.

Our data, spanning from 2018 to 2021, were collected from three main anaesthesiology training programs in Singapore. These data underwent preprocessing, which included standardization and cleaning to ensure consistency across different sources. Key variables affected by COVID-19, identified through a Delphi study, were central to our model's framework. These variables were systematically integrated into the SD model. The model's parameters were calibrated using this historical data, ensuring a realistic representation of the training environment's dynamics during the pandemic. In our SD model, we meticulously simulated the journey of residency candidates from admission to graduation. Candidates enter the system with an admission rate calibrated on historical data and progress through the training program, starting from R1 (first year of residency), through R3, and then finally to SR2 (second year of senior residency), before graduating as specialists. For each year of training, we set a pre-pandemic transfer rate to the next level, calibrated based on historical data. This rate represents the typical progression of residents under normal, pre-COVID-19 conditions. The model accounts for the impact of COVID-19 by adjusting these transfer rates based on various pandemic scenarios. This adjustment reflects the slowdown in resident progression due to pandemic-induced constraints, such as movement restrictions and altered training opportunities. A key feature of our model is the inclusion of a time variable that deactivates the COVID-19 impact after a pre-determined number of years. This duration varies according to the specific pandemic scenario being simulated, allowing us to study the short-term and long-term effects of the pandemic on residency progression. Central to our SD model is the use of the concept of stocks and flows. Stock variables, such as the number of active candidates in each year of training or the number of graduates in a specific year, are crucial. These are calculated using rate variables that account for changes in the stock over time. By incorporating feedback loops, our model simulates the dynamic behaviour of the system, capturing the complex interactions and dependencies inherent in residency training programs. The model's feedback loops are instrumental in understanding how changes in one part of the system (like admission rates or pandemic impact) influence other parts (such as graduation rates). This holistic view is vital for accurately simulating the residency training ecosystem, especially under the unprecedented strains imposed by the pandemic. Different durations of movement restrictions were simulated in the model, representing varying pandemic severities. This approach allowed us to explore a range of potential impacts on residency progression. We employed metrics such as average length of stay (ALOS) and ‘impact factor’ to quantify the delays. These metrics provided a clear measure of the pandemic's impact on residency progression, revealing significant delays, especially in the later years of training. A longer ALOS translates to a greater delay in progression. The ALOS should be a default of one year - any increase from that implies residents were delayed. For instance, a batch with an ALOS of 1.2 would require a mean of 1.2 years to graduate from that year of residency. Using the SHARP program data, we tested our model for accuracy and reliability. The outcomes aligned closely with observed delays, validating our model's effectiveness in predicting residency progression during pandemic conditions. For a more intuitive understanding, we included a detailed schematic (Figure 1) in our paper. Within this model, various durations of delay were also programmed into the software, where T (in years) was used to denote the duration of a circuit breaker. The T values we used ranged from 0 (best-case scenario) to 5 (worst-case scenario). The starting batch sizes for each residency year R1-SR2 in 2020 we used for our model were 12, 10, 10, 18, and 12, respectively.

Several assumptions were made in the development of the model. Firstly, it was assumed that residents who needed to repeat a training year would be able to make up for the delay within the next year and subsequently progress in their training. We also assumed that all residents would be able to pass the postgraduate exit exams at the first sitting and graduate after five completed years of training. Another assumption was that training delay during the pandemic was solely due to the implementation of a circuit breaker causing movement restrictions.

## Results

After the first phase of the study, we had a 100% response rate from the Delphi study (six out of six participants). Amongst the 32 variables, 20 variables had at least 80% agreement and were included in the development of our SD model.

As the minimum compulsory training requirements to be fulfilled for each accredited year vary, the effects of COVID-19 delays and hence, ALOS, would vary from one cohort to the next. Using the delays from the circuit breaker in 2020, the ALOS for the different batches ranged from 1.08 (R2) to 1.32 (SR1), indicating that residents in SR1 faced the greatest risk of delays following a circuit breaker. The ALOS for the other training years were 1.22 (R1) and 1.23 for R3 and SR2.

**Figure 2:**
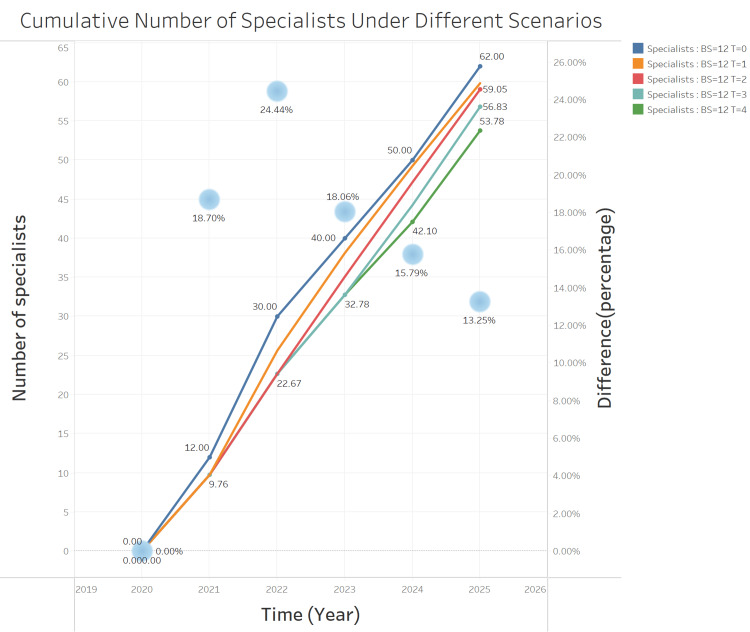
Cumulative number of residents graduating as specialists over time BS: batch size; T: time.

The model was also used to generate an estimate of the cumulative number of specialists graduating over the course of five years (i.e., 2020-2025), depending on the length of CB implemented. To generate this estimate, we utilized the model to predict the annual number of graduates for each year. We then simply summed these annual figures to determine the cumulative total over five years. This process did not require any reprogramming of our existing model, as it was capable of producing the necessary yearly graduate outputs that could be easily aggregated. As seen in Figure 2, the longer the duration of the circuit breaker, the lower the cumulative number of residents graduating as specialists.

**Figure 3:**
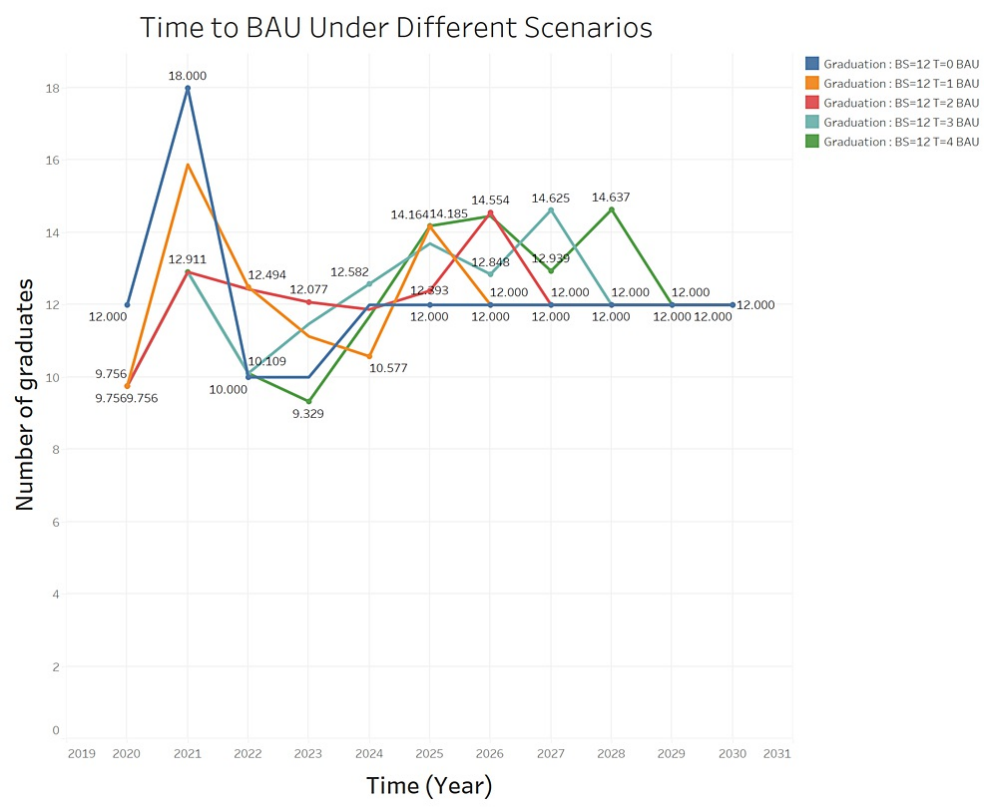
Time to business as usual (BAU) BS: batch size; T: time.

We also used this model to estimate the number of graduating specialists in any particular year for a period of up to 10 years following a period of CB as specified by the user. The process involved reprogramming the original model, treating the T = 0 base scenario as the standard business as usual (BAU) scenario. This step was followed by visualizing the number of graduates under various alternative scenarios. The time to return to the BAU condition was determined by identifying the point at which each plot intersected with the baseline scenario. This was done to gauge the time the residency program would take to return to BAU state. As expected, a longer duration of CB necessitated a longer duration of return to BAU. Our model was able to quantify this duration. As shown in Figure 3, a CB duration of four years would take the residency program approximately nine years to return to BAU and generate a baseline number of new specialists while a CB duration of one year only requires six years.

## Discussion

The use of SD modelling has increased substantially with many applications in medicine [10,11] and pandemic management [12-14]. However, to date, there is a paucity of literature on the use of SD modelling to predict delays in residency training and its impact on workforce provision.

We found that the SD model we created was able to predict the number of residents who would be delayed in any subsequent training year, the number of residents graduating as specialists, and the estimated duration required for the program to return to BAU depending on the duration of CB implemented. We have successfully validated the proposed simulation model through a comprehensive process. This involved ensuring that the model's structural elements, such as feedback loops, stocks, and flows, accurately represent the real-world system. We meticulously verified the parameters used in the model against real-world data from 2018 to 2021 from SHARP and reliable estimates to confirm their accuracy. Additionally, we conducted behavioural validation by comparing the model's output over time with actual system behaviour, affirming their alignment. Lastly, the model underwent a thorough expert review by subject matter experts, who validated its accuracy and realism, confirming its readiness for application in our studies.

Additionally, we have structured our simulation model in a manner that facilitates ease of implementation by others. The diagram in Figure 1 is designed to be intuitive and informative, allowing other researchers and practitioners to understand and adopt our model efficiently in their respective contexts. The proposed model can easily lend itself to modifications for the purposes of other residency training programs (e.g., with variable batch sizes and/or total duration of residency training). Other residency programs are able to input the variables specific to their own batches to generate accurate results. The model is accessible as an interactive use-friendly dashboard at "https://tinyurl.com/covmodel". Users are required to configure the model by specifying the duration of the program under study and entering the existing student population for each year within the program. Additionally, users must compute the average delay encountered by student cohorts due to the pandemic using their data, and then input these calculations into the model. Should there be a need, the differential equations and code underlying the model are available upon request.

As compared to pre-pandemic data, our data during the first year of the pandemic showed that movement restrictions between hospitals had the greatest impact on later residency training years (ALOS 1.23-1.32 for R3-SR2 vs. 1.08-1.22 for R1-R2). This can be partially explained by the residency structure itself, whereby a delay faced earlier in residency would allow more time for make-up compared to delays faced nearer the end of training. This may necessitate closer scrutiny of residents’ training schedules (especially senior residents) so that creative and dynamic schedule changes can be implemented to avoid delays in progression and graduation. Interestingly, the converse was shown in the study by Poyiadji et al. [15], which examined the impact of COVID-19 on radiology residency training. Their study showed a greater decrease in resident imaging interpretation amongst the R1s and R2s as compared to R3s and R4s. This was attributed to the R3s and R4s preferentially being accorded more clinical work and the R1s and R2s having more remote learning. The situation may be different in anaesthesia where our residents were not rostered for remote learning and efforts were made to prioritize continued clinical exposure for residents at each training site.

We also found that the training rotations most affected were the core subspecialty rotations (e.g., cardiothoracic, obstetrics, and paediatric anaesthesia), as these could only be done at speciality centres also subjected to restrictions. To overcome this, program directors can review alternative training sites and perhaps craft more flexible training schedules. This can include ‘front-loading’ training to cater for enforced quarantine periods or arranging ad-hoc alternatives in other training sites, as suggested by Nath et al. [16] in their report on the impact of COVID-19 on emergency medicine residency training.

The model prediction of the expected number of graduating specialists and the time required to return to BAU enabled a better understanding of the impact of the pandemic on our anaesthesia training program. As mentioned in the systematic review and analysis conducted by Chen et al. [17], extending residency programs or delaying specialist examinations would likely affect career planning and resident well-being. Our residency training program recognized this and proactively implemented mitigation strategies such as reshuffling of rotations for residents with expected delays. Still, the downstream effects on specialist throughput remain to be seen. Thus, predictions from this model can be used for planning manpower availability within training programs but also at a hospital or national level.

Limitations

The quantitative dataset of variables derived from the Delphi survey and used in developing this SD model may be different if new measures (not impacting movement restrictions) are implemented in subsequent CBs and other pandemics, thus reducing its generalizability. In addition, although it has been reported that movement restrictions, patient isolation, and cancellation of elective surgeries resulted in reduced case exposure [18,19], we did not use the case log requirement in the quantitative dataset for model input as we found that this was largely confounded by retrospective entries by residents. Still, qualitative assessment of case logs amongst residents did not suggest a significant impact by the CB, suggesting that most residents were able to gain exposure to a sufficient variety of cases in the various institutions. Lastly, our quantitative model was not able to account for any qualitative deficiencies in training due to the CB.

## Conclusions

SD modelling can be used to predict the impact of a pandemic on residency training if movement restrictions between training sites are implemented. Relevant stakeholders can thus implement mitigating measures early to minimize potential disruptions to residency progression and workforce throughput. With time and a greater dataset from various programs, the model can be further refined to improve the prediction accuracy for future cohorts of residents.

## Appendices

Table A1

**Table 1:** Delphi survey questionnaire with level of agreement amongst respondents ACLS: advanced cardiac life support.

Questions	Level of agreement (%)
"Do you feel that COVID-19 has affected a resident's ability to…."	
1. Complete junior residency within 36 months?	83
2. Have regular, scheduled didactic sessions (e.g., managing ambulatory surgical patients, managing geriatric patients, and clinical anaesthesiology)?	83
3. Have sufficient exposure to general/multi-system anaesthesia?	83
4. Fulfil four months of distinct and progressive rotations in critical care medicine?	67
5. Fulfil at least one month in an acute perioperative pain rotation.	50
6. Fulfil at least one month of managing inpatients and outpatients in a chronic pain rotation?	83
7. Fulfil at least one month of regional anaesthesia as part of pain medicine?	83
8. Fulfil two distinct one-month rotations in obstetric anaesthesia?	83
9. Fulfil two distinct one-month rotations in paediatric anaesthesia?	83
10. Fulfil two distinct one-month rotations in neuroanaesthesia?	83
11. Fulfil two distinct one-month rotations in cardiothoracic anaesthesia?	67
12. Fulfil one month in a pre-operative evaluation clinic?	17
13. Fulfil two weeks managing patients in the post-anaesthesia care unit?	17
14. Maintain current ACLS certification?	83
15. Gain experience in managing patients undergoing vaginal delivery, including high-risk obstetric cases?	67
16. Gain experience in managing patients undergoing caesarean sections?	83
17. Gain experience in managing paediatric patients younger than 12 years of age?	100
18. Gain experience in managing patients undergoing cardiac surgery (including those undergoing cardiopulmonary bypass)?	67
19. Gain experience in managing patients undergoing open or endovascular procedures on major vessels (e.g., carotid surgery, intra-thoracic/-abdominal major vascular surgery) (not including surgery for vascular access)?	83
20. Gain experience in managing patients undergoing non-cardiac intra-thoracic surgery (e.g., pulmonary surgery and mediastinal surgery)?	100
21. Gain experience in managing patients undergoing intracerebral procedures (including patients undergoing endovascular procedures, and surgeries involving an open cranium)?	67
22. Gain experience in managing patients undergoing procedures involving placement of epidural catheters (including caesarean sections and combined spinal-epidural)?	83
23. Gain experience in managing patients undergoing surgery for complex, life-threatening injuries?	83
24. Gain experience in managing patients with severe burns covering more than 20% of the body surface area?	67
25. Gain experience in managing patients undergoing surgeries involving peripheral nerve blocks?	67
26. Gain experience in managing new patients referred for management of acute, chronic, or cancer-related pain disorders (including experience in interventional pain procedures)?	83
27. Gain experience in advanced airway techniques (e.g., fibreoptic intubation, lung isolation, and double-lumen tube placement)?	100
28. Gain experience in the placement of central venous catheters and ultrasound-guided vascular catheters?	33
29. Gain experience in the placement of pulmonary artery catheters or other cardiac output monitoring devices?	50
30. Gain experience in performing transesophageal echocardiography (TEE) or receiving sufficient didactic teaching on the techniques and interpretation?	100
31. Gain experience in electroencephalography (EEG), processed EEG, or evoked potential monitoring or receive sufficient didactic instruction in these?	67
32. Complete an academic project?	83
